# Impact of type 2 diabetes mellitus on results in the animal naming test in patients with and without liver cirrhosis

**DOI:** 10.1371/journal.pone.0316490

**Published:** 2025-02-06

**Authors:** Eva Maria Schleicher, Julia Tuchscher, Matthias Weber, Peter Robert Galle, Marcus-Alexander Wörns, Simon Johannes Gairing, Christian Labenz

**Affiliations:** 1 Department of Internal Medicine I, University Medical Center of the Johannes Gutenberg-University, Mainz, Germany; 2 Cirrhosis Center Mainz (CCM), University Medical Center of the Johannes Gutenberg-University, Mainz, Germany; 3 Department of Gastroenterology, Hematology, Oncology and Endocrinology, Klinikum Dortmund, Dortmund, Germany; Medizinische Fakultat der RWTH Aachen, GERMANY

## Abstract

**Introduction and objectives:**

Diabetes mellitus is a common comorbidity in patients with cirrhosis and is associated with the development of hepatic encephalopathy (HE) and cognitive dysfunction. The simplified Animal Naming Test (S-ANT1) has been established for detecting minimal HE (MHE). It is currently unknown whether S-ANT1 results are affected by diabetes mellitus in patients with and without cirrhosis.

**Materials and methods:**

This study analyzed data from 268 patients with cirrhosis without signs of HE ≥ 1. MHE was defined using the psychometric hepatic encephalopathy score (PHES). All patients were also tested with S-ANT1. 14 patients with diabetes mellitus and diabetic foot syndrome but no cirrhosis, as well as 37 healthy controls, were also tested with S-ANT1 and served as controls.

**Results:**

Type 2 diabetes mellitus was present in 79 (29.5%) patients with cirrhosis and MHE according to PHES was detected in 81 (30.2%) patients. In the total cohort, results in S-ANT1 did not differ between patients with and without diabetes mellitus (19 vs. 20 animals, p = 0.108). In multivariable logistic regression analysis, the only variables independently associated with performance in S-ANT1 were PHES-MHE, school education, sodium, and age, while diabetes mellitus was not. Patients with diabetic foot syndrome but no cirrhosis performed poorer in S-ANT1 compared to healthy controls, while patients with cirrhosis and MHE performed poorer than patients with diabetic foot syndrome.

**Conclusion:**

S-ANT1 seems to be usable for screening for MHE in patients with cirrhosis and type 2 diabetes mellitus, while one might be more cautious when interpreting results in patients with diabetes-related complications.

## Introduction

Hepatic encephalopathy (HE) is a frequent complication in patients with cirrhosis and is associated with a poor prognosis as well as severe consequences for daily life [1]. The lowest grade of HE is minimal HE (MHE). MHE is below the obvious clinical detection level and therefore specialized tests are needed to establish a diagnosis [2]. Although subclinical, MHE is a huge burden for patients and caregivers given that it is associated with poorer health-related quality of life, car accidents, and car crashes [3,4].

The diagnostic armamentarium to detect MHE includes many different tests, such as the portosystemic encephalopathy syndrome test, which yields the psychometric hepatic encephalopathy score (PHES). PHES has been developed for the detection of MHE more than 20 years ago, but remains the gold standard [5]. Beyond PHES, there are also other tests available, such as the determination of the critical flicker frequency, the inhibitory control test, or the Stroop EncephalApp [6]. Although well established and readily available, these tests have in common that they are rarely used in daily routine most likely due to their time-consuming nature or additional costs [7]. In 2017 the Animal Naming Test (ANT) was introduced to the field and subsequently validated as a rapid test for diagnosing MHE within one minute [8,9]. However, one downside of ANT might be that it especially tests word fluency and memory, cognitive domains that are typically not affected by MHE. Therefore, results in ANT could be significantly affected by concomitant comorbidities.

One of the most frequent comorbidities in patients with cirrhosis is diabetes mellitus with a prevalence ranging between 22–40% in patients with cirrhosis [10,11]. While diabetes mellitus has been previously linked to a higher risk of MHE as well as OHE [12], the association between diabetes mellitus and cognitive deficits is also well-established [13–15]. In patients with type 2 diabetes mellitus, cognitive alterations mainly affect learning ability, memory as well as mental flexibility, and speed [15–18]. Additionally, diabetes mellitus is associated with a clinically relevant risk increase for dementia [15,19]. All the aforementioned factors might influence the performance of ANT. This leads to the hypothesis that performance in ANT might be clinically relevant influenced by underlying diabetes mellitus and that ANT may not be suitable for diagnosing MHE in patients with cirrhosis and diabetes mellitus. Therefore, his study aimed to investigate the impact of diabetes mellitus on results in ANT in patients with and without liver cirrhosis.

## Methods

### Patient cohorts

This study was conducted at the Cirrhosis Center Mainz (CCM) which is part of the Department of Internal Medicine I at the University Medical Center Mainz, Germany. Patients were recruited between 10^th^ of July 2017 and 31^st^ of December 2023. All patients were either recruited in the outpatient department or during elective hospitalization. Accepted reasons for elective hospitalization were either to perform a liver biopsy, paracentesis (without evidence of spontaneous bacterial peritonitis), esophagogastroduodenoscopy with expected band ligation, or evaluation for liver transplantation. A subgroup of this cohort (n = 143) has been previously used for the validation of S-ANT1 in German patients with cirrhosis [9].

Disease characteristics were documented after a review of the electronic medical patient chart. Diagnosis of cirrhosis was established by histology or a combination of conclusive appearance in ultrasound, radiological imaging, endoscopic features of portal hypertension, and medical history. Patients were not approached for this study, if they fulfilled one of the exclusion criteria. These included an active infection, ongoing alcohol abuse during the last three months, use of psychotropic drugs or opioids, the presence of pre-terminal comorbidities affecting the heart or the lungs, hepatocellular carcinoma or other malignancies, presence of a transjugular intrahepatic portosystemic shunt (TIPSS), neurological comorbidities (i.e. dementia or history of stroke), history of recent head trauma and not sufficient German language skills.

A history of OHE was not treated as an exclusion criterion. However, patients had to be stable for at least six weeks and had to be on stable treatment with lactulose / rifaximin.

As controls, 14 patients with diabetes mellitus type 2 and diabetic foot syndrome were also recruited from the outpatient clinic of the Department of Endocrinology, which is a part of the Department of Internal Medicine I at the University Medical Center Mainz. Those patients had no evidence or signs of liver cirrhosis and the same exclusion criteria as mentioned above applied.

Additionally, 37 healthy volunteers were recruited as another control group (median age: 56 years (IQR 50, 63), male sex: 18 (48.6%), school education in years: 10 (IQR 10, 12.5). Data from these controls were previously published in the validation of S-ANT1 in German patients [9].

### Diagnosis of HE

Clinical signs of HE (HE ≥ 1) were ruled out by a hepatologist. HE was graded according to the West Haven criteria as recommended in the guidelines [2].

Diagnosis of MHE was established using the portosystemic encephalopathy (PSE) syndrome test, which yields the psychometric hepatic encephalopathy score (PHES). Testing was done following a structured protocol in a quiet and lighted room and was never performed on the same day as any other intervention. A score < -4 was considered pathological according to German norms [5].

As a second test for cognitive function, we tested all patients of the study cohorts with the Animal Naming Test (ANT). For the diagnosis of MHE, the ANT was first described by Campagna et al. [8]. In brief, patients are asked to name as many animals as possible in one minute, while repeats and errors are excluded. This yields the score of the ANT. It is known that ANT is influenced by age and school education. Therefore, we calculated the simplified ANT (S-ANT1), which accounts for age and education and has also been developed and published by Campagna et al. [8]. Results in S-ANT were considered pathological for < 20 enumerated animals, according to the German validation study [9].

### Ethics

Written informed consent was obtained from all participants. The study was conducted in accordance following the ethical guidelines of the 1975 Declaration of Helsinki and its later amendments. The study was approved by the ethics committee of the Landesärztekammer Rheinland-Pfalz (Nr. 837.232.17 [11066]).

### Statistical analysis

Quantitative data are presented as medians with interquartile ranges (IQR). To compare quantitative variables between the two groups, we used an unpaired t-test or the Mann-Whitney U Test. Testing for normal distribution of quantitative variables was conducted using the Shapiro-Wilk test. Categorical variables are presented as frequencies and percentages. For comparison between two or more groups, a chi-square test was applied. Differences between three or more with metric data were evaluated with an ordinary one-way ANOVA with Tukey’s multiple-comparisons test. For correlation analyses between HbA1c and S-ANT1, we used Spearman’s rank correlation. To identify variables independently associated with the performance in S-ANT1, multivariable linear regression models were conducted using a stepwise variable selection procedure.

To define statistically relevant deviations from the respective null hypotheses, the level of p-values was set at 0.05. When interpreting all results, it is important to note that the analyses are exploratory and not adjusted for multiple testing.

Data were analyzed using IBM SPSS Statistic Version 27.0 (Armonk, NY: IBM Corp.) and GraphPad Prism Version 8.0.2 (GraphPad Software, California, US).

## Results

### Demographics and baseline characteristics of patients with liver cirrhosis

In total, 309 patients were recruited. 24 patients were excluded from the following analyses due to the presence of HE grade 1 according to the West Haven criteria and 17 patients were not able to perform ANT because they were not speaking German fluently, resulting in a cohort of 268 patients with cirrhosis (see S1 File). The baseline characteristics of this cohort are displayed in **Table 1**. Diabetes mellitus was present in 79 (29.5%) patients. All patients with diabetes had diabetes mellitus type 2. Of the patients with diabetes mellitus, 29.1% were treated with metformin and 51.9% with insulin. MHE according to PHES was detected in 81 (30.2%) patients. Median S-ANT1 in the total cohort was 20 (IQR 16; 24) animals. Of note, outpatients had significantly better results in S-ANT1 than inpatients (21 vs. 18, p < 0.001).

**Table 1:** Demographics and clinical characteristics of the total cohort, patients with and without diabetes at the time of study inclusion.

Variable		All patients n = 268	Patients with diabetes n = 79	Patients without diabetes n = 189	p-value^#^
Age, years (IQR)		60 (53; 65)	63 (58; 67)	58 (51; 65)	<0.001^+^
Male gender, n (%)		157 (58.6)	49 (62.0)	108 (57.1)	0.459*
School education, years (IQR)		10 (9; 10)	9 (9; 10)	10 (9; 12)	0.005^+^
Aetiology	Alcohol, n (%) Viral hepatitis, n (%) MASLD, n (%) Cholestatic/ Autoimmune, n (%) Other/mixed, n (%)	106 (39.6) 45 (16.8) 36 (13.4) 30 (11.2) 51 (19.0)	23 (29.1) 7 (8.9) 24 (30.4) 4 (5.1) 21 (26.6)	83 (43.9) 38 (20.1) 12 (6.3) 26 (13.8) 30 (15.9)	<0.001*
Median MELD score (IQR)		10 (8; 14)	10 (8; 13)	11 (8; 14)	0.598^+^
Child-Pugh A/B/C, n (%)		148 / 100 / 20 (55.2 / 37.3 / 7.5)	45 / 31 / 3 (60.0 / 39.2 / 3.8)	103 / 69 / 17 (54.5 / 36.5 / 9.0)	0.335*
History of ascites, n (%)		155 (57.8)	43 (54.4)	112 (59.3)	0.465*
History of OHE, n (%)		37 (13.8)	15 (19.0)	22 (11.6)	0.112*
Sodium, mmol/l (IQR)		138 (136; 140)	137 (135; 139)	138 (137; 140)	0.007^+^
Albumin, g/l (IQR)		34 (29; 38)	35 (31; 38)	34 (29; 38)	0.426^
Bilirubin, mg/dl (IQR)		1.2 (0.8; 2.0)	1.1 (0.7; 1.6)	1.2 (0.8; 2.1)	0.070^+^
Creatinine, mg/dl (IQR)		0.9 (0.8; 1.0)	0.9 (0.8; 1.2)	0.9 (0.8; 1.0)	0.027^+^
INR (IQR)		1.2 (1.1; 1.4)	1.2 (1.1; 1.4)	1.2 (1.1; 1.4)	0.184^+^
Platelets, /nl (IQR)		121 (80; 172)	118 (80; 172)	122 (81; 173)	0.870^+^
Ammonia, μmol/l (IQR)*		45 (36; 58)	47 (37; 56)	45 (36; 60)	0.569^+^
HbA1c, % (IQR)**		5.3 (4.9; 6.0)	6.7 (5.9; 8.0)	5.1 (4.7; 5.5)	<0.001^+^
MHE, n (%)		81 (30.2)	33 (41.8)	48 (25.4)	0.008
S-ANT1 (IQR)		20 (16; 24)	19 (16; 22)	20 (16; 24)	0.108^+^

Data are expressed as medians and interquartile ranges or as frequencies and percentages; MASLD, metabolic dysfunction-associated steatotic liver disease, metALD. Metabolic dysfunction overlapping with alcoholic liver disease, MELD, model for end-stage liver disease; MHE, minimal hepatic encephalopathy; S-ANT1, simplified animal naming test; CFS, clinical frailty scale.

*available in 242 patients

**available in 249 patients

^#^comparison between patients with and without diabetes mellitus

^+^ Mann-Whitney U Test

^unpaired t-test

* chi-square test.

### Influence of diabetes mellitus on results in S-ANT1 in patients with cirrhosis

**Fig 1:**
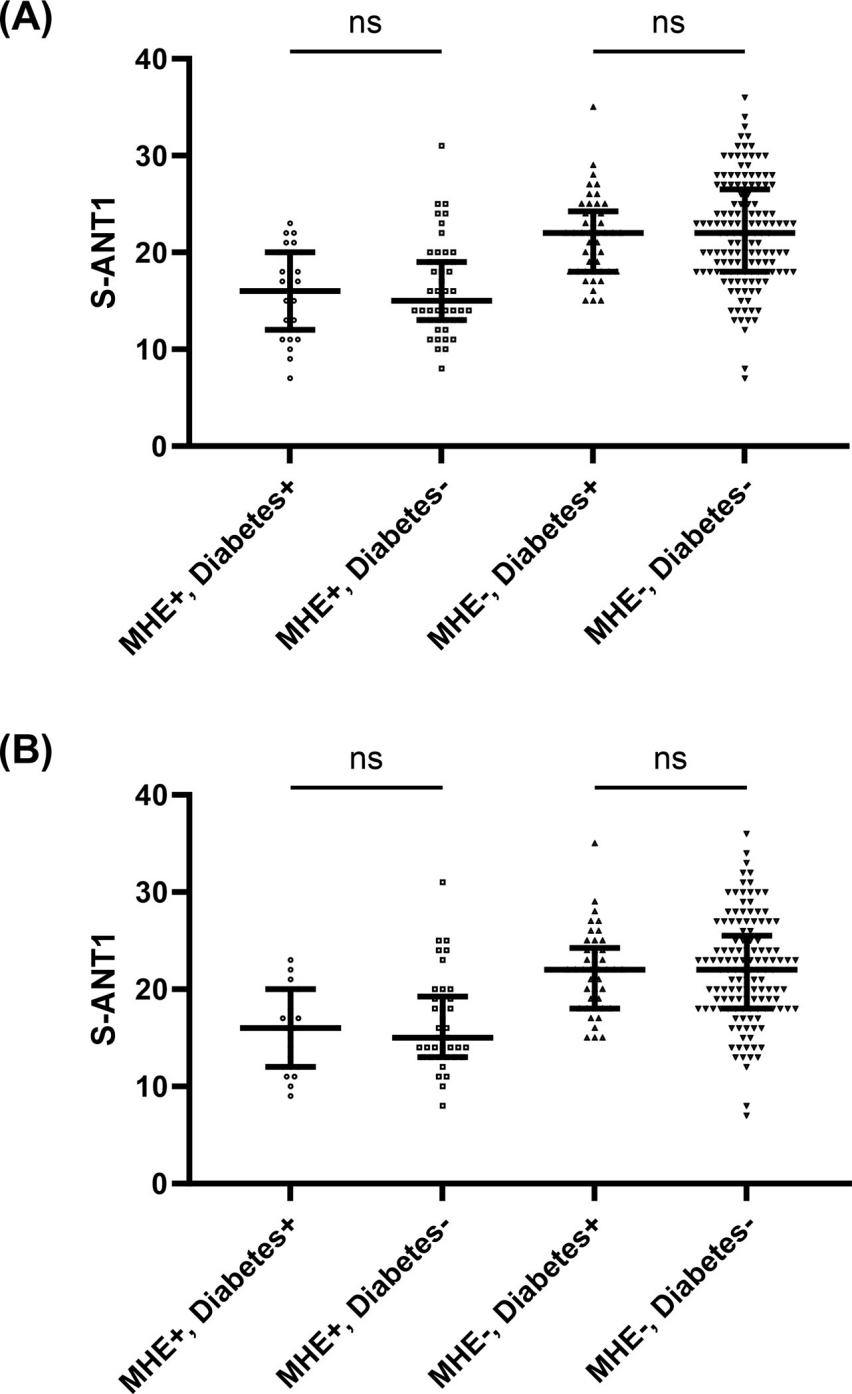
Comparison of results in S-ANT1 in patients with cirrhosis with and without MHE, either with or without diabetes. A displays median and interquartile ranges of results in S-ANT1 in subgroups of the total cohort. B displays median and interquartile ranges of results in S-ANT1 in subgroups of patients without a history of overt hepatic encephalopathy. MHE: Minimal hepatic encephalopathy, S-ANT1: Simplified animal naming test.

**Fig 2:**
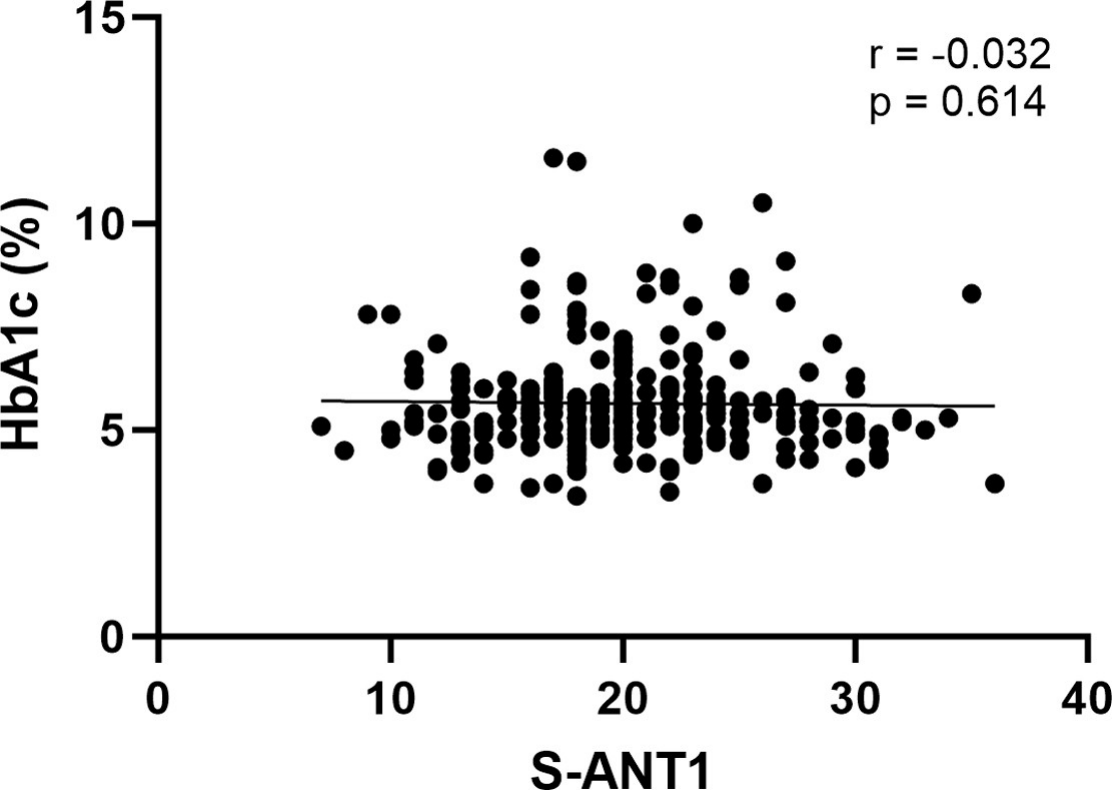
Correlation between HbA1c values and results in S-ANT1 in patients with liver cirrhosis. S-ANT1: Simplified animal naming test.

Comparisons of baseline characteristics between patients with cirrhosis with and without diabetes mellitus are displayed in **Table 1**. In brief, both groups differed in terms of age, years of school education, serum sodium, creatinine, HbA1c and presence of MHE according to PHES. In the total cohort, results in S-ANT1 did not differ between patients with and without diabetes mellitus (19 vs. 20 animals, p = 0.108). In subgroup analyses, stratified by MHE status, there was no difference in results in S-ANT1 when comparing patients with and without diabetes mellitus (**Fig 1A**). This finding remained unchanged in another analysis after excluding all patients with a history of OHE (**Fig 1B**). There was also no relevant correlation between glycemic control during the past weeks, as expressed by HbA1c, and results in S-ANT1 (**Fig 2**).

The discriminative ability of S-ANT1 to distinguish between patients with and without MHE according to PHES in the total cohort of patients with cirrhosis was good with an area under the curve (AUC) of 0.80 (95% CI 0.75, 0.86). There was no significant difference in the discriminative ability of S-ANT1 to detect MHE according to PHES in patients with (AUC 0.83, 95% CI 0.75, 0.92) or without diabetes mellitus (AUC 0.79, 95% CI 0.71, 0.87).

To identify variables associated with performance in S-ANT1 in the total cohort of patients with cirrhosis, we built multivariable linear regression models based on a stepwise variable selection procedure. Here, the only variables independently associated with performance in S-ANT1 were MHE, school education, sodium and age (**Table 2**). This finding did not change when including the location of testing (in the outpatient setting vs. in the inpatient setting) in the model (**S1 Table**). The same variables remained independently associated with performance in S-ANT1 in a subgroup analysis after excluding all patients with a history of OHE (**Table 2**). Of note, diabetes mellitus was not associated with performance in S-ANT1 (**Table 2**) in multivariable analyses and neither was HbA1c (p = 0.219).

**Table 2:** Variables associated with the results in S-ANT1 using multivariable linear regression models.

	Regression coefficient (95% CI)	β	p-value
Model 1: total cohort (n = 268)			
**MHE**	-4.84 (-6.13; -3.55)	-0.40	<0.001
**School education**, years	0.66 (0.32; 1.00)	0.20	<0.001
**Sodium,** mmol/l	0.26 (0.09; 0.44)	0.16	0.004
**Age,** years	-0.08 (-0.13; -0.02)	-0.15	0.008
**Model 2: patients without a history of OHE (n = 231)**			
**MHE**	-4.68 (-6.16; -3.21)	-0.37	<0.001
**School education,** years	0.57 (0.18; 0.95)	0.17	0.004
**Sodium,** mmol/l	0.27 (0.06; 0.48)	0.15	0.012
**Age,** years	-0.09 (-0.15; -0.03)	-0.17	0.005

The multivariable linear regression models were performed with a stepwise variable selection procedure.

95% CI, 95% confidence interval; MHE, minimal hepatic encephalopathy; OHE, overt hepatic encephalopathy.

Model 1: R2 0.303; variables not reaching significance were: Diabetes (p = 0.24), gender (p = 0.81), MELD (p = 0.19), alcoholic cirrhosis (p = 0.07), albumin (p = 0.70), history of OHE (p = 0.65), history of ascites (p = 0.15), platelets (p = 0.72).

Model 2: R2 0.260, variables not reaching significance were: Diabetes (p = 0.32), gender (p = 0.79), MELD (p = 0.38), alcoholic cirrhosis (p = 0.08), albumin (p = 0.88), history of ascites (p = 0.07), platelets (p = 0.61).

### Comparison of results in S-ANT1 between patients with liver cirrhosis, patients with diabetic foot syndrome and no cirrhosis, and healthy controls

**Fig 3:**
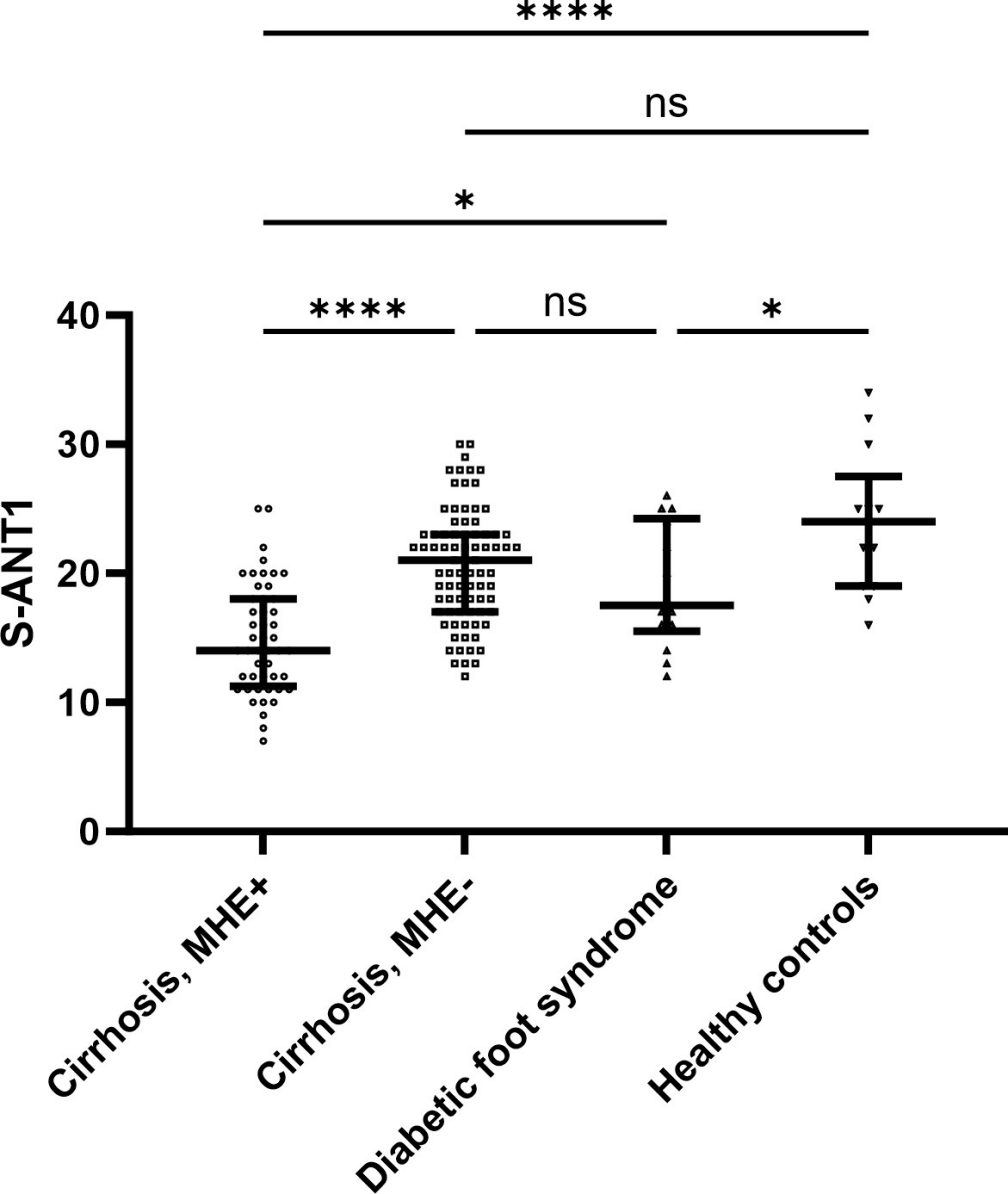
Comparison of results in S-ANT1 in patients with cirrhosis with and without MHE, patients with diabetic foot syndrome without cirrhosis and healthy controls. *p < 0.05, ****p < 0.0001 MHE: Minimal hepatic encephalopathy, S-ANT1: Simplified animal naming test.

As a comparison to patients with liver cirrhosis, we also recruited 14 patients with diabetic foot syndrome as a diabetes-related complication without cirrhosis. Additionally, we recruited 37 healthy controls. However, given that the 14 patients with diabetic foot syndrome were significantly older than the healthy controls, we only included 13 healthy controls with an age > 60 years. The same case selection (> 60 years) was performed to get a comparable subgroup of patients with cirrhosis (n = 129). Characteristics of patients with diabetic foot syndrome, healthy controls and the subgroup of patients with cirrhosis are displayed in **S2 Table**. Patients with diabetic foot syndrome performed poorer in S-ANT1 compared to healthy controls (**Fig 3**). Patients with cirrhosis and MHE performed poorer in S-ANT1 compared to patients with diabetic foot syndrome, while performance in S-ANT1 was comparable between patients with cirrhosis without MHE and patients with diabetic foot syndrome (**Fig 3**). Of note, 8/14 patients with diabetic foot syndrome had an S-ANT1 < 20 animals (cut-off for MHE according to German norms).

## Discussion

Diabetes mellitus is a common comorbidity in patients with cirrhosis and is associated with the development of hepatic encephalopathy (HE) and cognitive dysfunction [12,15]. Some of these mental changes overlap with dysfunctions caused by HE and may hamper the interpretation of MHE tests. In this study, we found no clinically relevant impact of underlying type 2 diabetes mellitus on results in S-ANT1 in patients with liver cirrhosis. This was even stressed by a lack of a correlation between glycemic control, as expressed by HbA1c, and results in S-ANT1 in patients with liver cirrhosis. However, we found mostly poor results in S-ANT1 below the German cut-off for detection of MHE in cirrhosis in patients with severe diabetes and diabetic foot syndrome without evidence of cirrhosis, suggesting that diabetes-related complications might influence results in S-ANT1.

In the Western world, metabolic dysfunction-associated steatotic liver disease (MASLD) is the strongest rising etiology in hospitalized patients with decompensated cirrhosis [20]. This results in a rising prevalence of diabetes mellitus in patients with cirrhosis, which might both have additive effects on cognitive dysfunction. The prevalence of MHE roughly ranges between 25–50% depending on the Child-Pugh stages, while the prevalence of cognitive dysfunction caused by diabetes mellitus is estimated to be as high as 45% in patients without cirrhosis [13,21]. Evidently, ANT is unspecific and has been adapted from other cognitive disorders in the field of HE [8]. A recent study by Tapper et al. analyzing more than 6,000 subjects from the US Health and Retirement Survey found performance in ANT not being linked to cirrhosis but especially frailty [22]. However, it has to be acknowledged that the median age of this cohort was 75 years, and the prevalence of cirrhosis was only 1.3%. Nevertheless, these data impressively indicate that the results of ANT have to be interpreted in the context of their use and that underlying conditions might influence test results heavily.

To our own surprise, diabetes mellitus was not associated with poorer results in S-ANT1 in our current study and we only found a clinically relevant decline in performance in S-ANT1 in patients with diabetic foot syndrome (without cirrhosis). This finding is of importance for routine clinical practice because S-ANT1 seems to be applicable for the detection of MHE in patients with cirrhosis and compensated diabetes mellitus without any restrictions. Moreover, the diagnostic accuracy seems to be comparable to patients with cirrhosis and no diabetes.

Diabetes mellitus, especially uncontrolled or poorly controlled, is associated with cognitive alterations mainly affecting executive functions, attention, processing speed, memory, as well as mental flexibility and speed [23]. Therefore, it seemed to be a reasonable hypothesis that the presence of diabetes mellitus in general might influence results in S-ANT1. Our current study does not give any mechanistic insight, and hence, we are only able to hypothesize potential explanations for our findings. One potential interpretation might be that the presence of MHE outweighs the effects of cognitive alterations due to diabetes mellitus on performance in S-ANT1 in the investigated cohort of patients with cirrhosis. However, even patients with neither MHE nor diabetes performed comparable to patients without MHE but with diabetes. This could imply that cognitive disorders affecting the skills needed to sufficiently perform ANT are influenced only in more advanced stages of diabetes mellitus. Here, our findings are in line with a study comparing patients with type 2 diabetes mellitus with and without mild cognitive impairment [24]. In this study, patients with mild cognitive impairment caused by diabetes mellitus performed similarly in ANT compared to patients without cognitive impairment. Similar findings were made in a study comparing patients with type 1 diabetes mellitus with healthy controls [25]. Bortolotti et al. found no difference in ANT between both groups, while the authors especially detected a slowing in psychomotor speed in patients with a history of previous hypoglycaemic episodes or coma. Consequently, this is also in line with our current findings demonstrating that diabetes mellitus without diabetes-related complications might not impact testing with ANT.

This study has limitations that have to be acknowledged. Firstly, the group of patients without cirrhosis and with diabetic foot syndrome is comparably small. However, the group was sufficiently large to demonstrate significant deviations in S-ANT1 from healthy controls indicating cognitive deficits in this group. Secondly, this study lacks information on insulin resistance, e.g. by measuring a Homeostasis Model Assessment-Insulin Resistance (HOMA-IR), which might have given additional pathophysiological insight. Additionally, it has to be acknowledged that HbA1c, which was used as a surrogate for glycemic control during the past weeks in this study, does not reflect glycemic control for the whole time since diabetes onset. This is an important limitation, given that cognitive deficits in diabetic patients are expected to depend on the sum of vascular lesions as well as hypoglycemic and hyperglycemic episodes that occurred in the past. Thirdly, we did not actively search for complications of diabetes mellitus in our patients with cirrhosis. Therefore, we are unable to establish a more granular stratification of diabetes mellitus besides HbA1c in patients with cirrhosis. Last, we did not assess glucose levels at the time of cognitive testing following a predefined protocol. Therefore, we cannot exclude a potential bias of hypo- or hyperglycaemia on test results. Additionally, HbA1c was not available in each patient and in these patients the diagnosis of diabetes was assessed by asking the patient and analyzing the electronic patient chart. However, it might be possible that some of these patients might have suffered from undetected diabetes mellitus.

In conclusion, we found no clinically relevant impact of underlying type 2 diabetes mellitus on results in S-ANT1 in patients with liver cirrhosis. However, patients with diabetic foot syndrome but no cirrhosis had mostly impaired results in S-ANT1. Following these findings, S-ANT1 seems to be usable for screening for MHE in patients with cirrhosis and compensated forms of diabetes mellitus, while one might be more cautious when interpreting the results in patients with severe complications from diabetes. These findings have to be validated in a future prospective multicenter study.

## Supporting information

S1 File Flow-chart of the study. (PDF)

S1 Table Variables associated with the results in S-ANT1 using multivariable linear regression models. (PDF)

S2 Table Demographics and clinical characteristics of the cirrhosis cohort (aged > 60 years), patients with diabetes and diabetic foot syndrome without cirrhosis, and healthy controls (aged > 60 years). (PDF)

## Abbreviations

ANT: Animal naming test
AUC: Area under the curve
CCM: Cirrhosis Centre Mainz
CHE: covert hepatic encephalopathy
CI: confidence interval
CP: Child-Pugh
HE: hepatic encephalopathy
HCC: hepatocellular carcinoma
S-ANT1: simplified animal naming test
HOMA-IR: Homeostasis Model Assessment-Insulin Resistance
INR: international normalized ratio
IQR: interquartile range
MASLD: metabolic dysfunction-associated steatotic liver disease
MELD: Model of end-stage liver disease
MHE: minimal hepatic encephalopathy
OHE: overt hepatic encephalopathy
OR: odds ratio
PHES: Psychometric Hepatic Encephalopathy Score
PSE: portosystemic encephalopathy
TIPSS: transjugular intrahepatic portosystemic shunt
