# Supplementary figures and images for: Impact of type 2 diabetes mellitus on results in the animal naming test in patients with and without liver cirrhosis
Source: PLoS One. 2025 Feb 6;20(2):e0316490. doi: 10.1371/journal.pone.0316490 (PMC11801616; doi:10.1371/journal.pone.0316490)

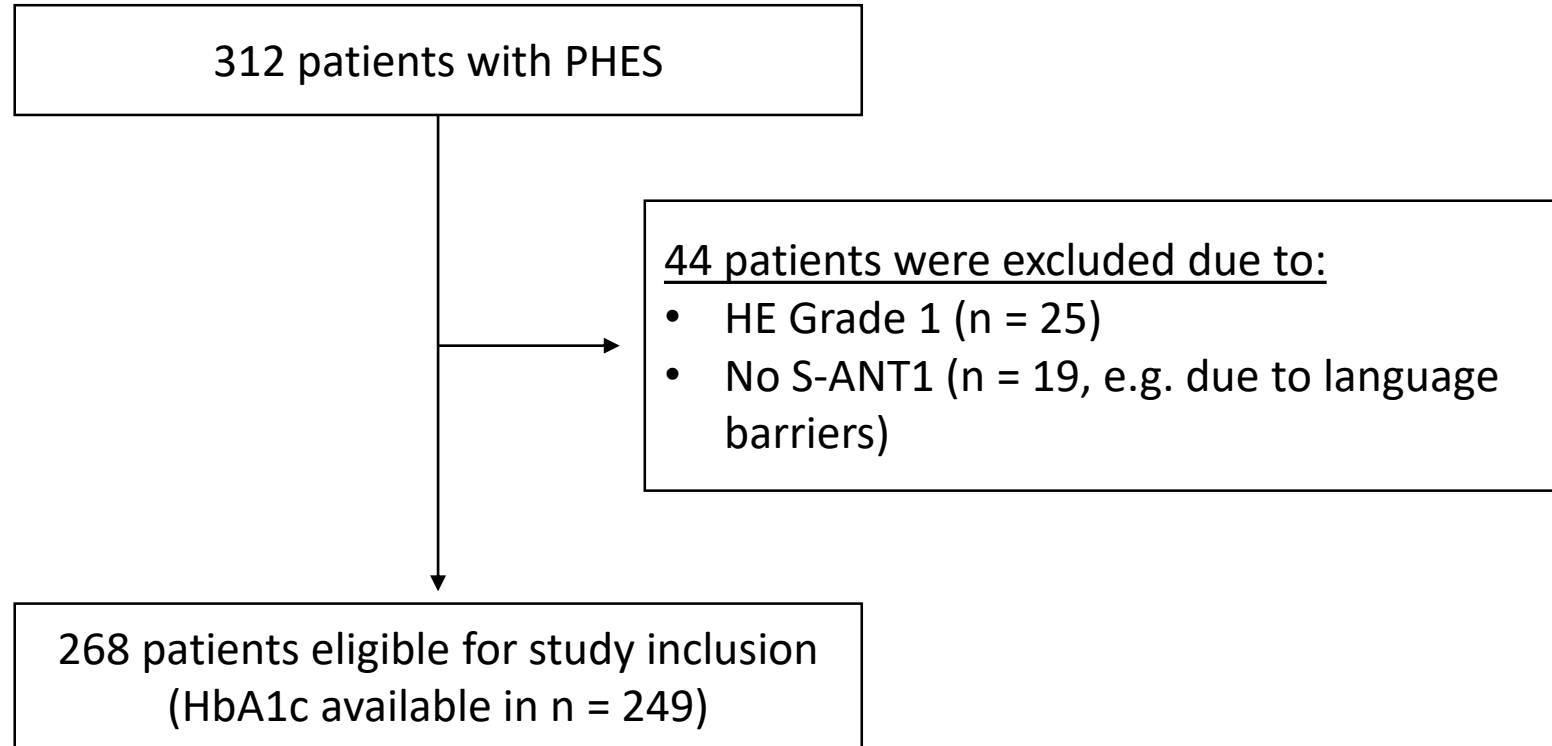

Supplement: S1 File — (PDF) [file pone.0316490.s001.pdf]
