# Supplementary material for: Impact of type 2 diabetes mellitus on results in the animal naming test in patients with and without liver cirrhosis
Source: PLoS One. 2025 Feb 6;20(2):e0316490. doi: 10.1371/journal.pone.0316490 (PMC11801616; doi:10.1371/journal.pone.0316490)
Supplement: S1 Table — (PDF) [file pone.0316490.s002.pdf]

Supplementary table 1. Variables associated with the results in S-ANT1 using multivariable linear regression models.

	Regression coefficient (95% CI)	β	p-value
total cohort (n = 268)			
**MHE**	-4.67 (-5.96, -3.38)	-0.38	<0.001
**School education**, years	0.68 (0.34, 1.02)	0.20	<0.001
**Sodium,** mmol/l	0.22 (0.03, 0.40)	0.13	0.022
**Age,** years	-0.08 (-0.14, -0.02)	-0.15	0.005
**Tested in the inpatient setting**	-1.19 (-2.41, 0.04)	-0.10	0.058

The multivariable linear regression model was performed using predefined variables.

R2: 0.310

95% CI, 95% confidence interval; MHE, minimal hepatic encephalopathy; OHE, overt hepatic encephalopathy
