# Supplementary material for: Impact of type 2 diabetes mellitus on results in the animal naming test in patients with and without liver cirrhosis
Source: PLoS One. 2025 Feb 6;20(2):e0316490. doi: 10.1371/journal.pone.0316490 (PMC11801616; doi:10.1371/journal.pone.0316490)
Supplement: S2 Table — (PDF) [file pone.0316490.s003.pdf]

Supplementary table 2. Demographics and clinical characteristics of the cirrhosis cohort (aged > 60 years), patients with diabetes and diabetic foot syndrome without cirrhosis, and healthy controls (aged > 60 years).

Variable	Patients with cirrhosis n = 129	Patients with diabetic foot syndrome n = 14	Healthy controls n = 13	p-value (ANOVA between groups)
Age, y (IQR)	65 (63; 72)	72 (73; 80)	68 (63; 74)	0.082
Male gender, n (%)	75 (58.1)	13 (92.9)	5 (38.5)	0.011
School education, y (IQR)	9 (8; 10)	10 (8; 11)	10 (8; 10)	0.699
S-ANT1 (IQR)	18 (14; 22)*	18 (16; 24)^	24 (19; 28)*^	0.002

Data are expressed as medians and interquartile ranges or as frequencies and percentages;
*significant difference between patients with cirrhosis and healthy controls
^significant difference between patients with diabetic foot syndrome and healthy controls
